# On a Case of Abnormal Position of the Viscera

**Published:** 1849-08

**Authors:** G. I. Knight

**Affiliations:** Abingdon


*On a Case of Abnormal Position of the Viscera.* By G. I. Knight, Esq., Abingdon.—Thomas W——, aged eighty-six, died suddenly October 20th, 1848.

*Post-mortem,* Oct. 21st.—On opening the thorax the heart was found much more to the right side than usual, the apex being opposite the middle of the sternum, or, perhaps, even rather to the right side of it. The heart itself was very large and flabby, and the valves partially ossified. Lungs, posteriorly, very much gorged with blood, approaching the state termed apoplexy of the lung; otherwise quite healthy. The liver quite healthy, placed in a position exactly the reverse of natural, the larger lobe, with the gall-bladder in front of it, occupying the left hypochondrium, while the spleen was in the right. The cæcum, with its appendix vermiformis, was situated in the left iliac fossa, while the sigmoid flexure of the colon occupied the right. The viscera healthy, excepting that there existed several cysts containing a pellucid albuminous fluid; one, about the size of a small walnut, in the left kidney; two on the under surface of the liver, and a small one in the right kidney.

The individual in whom this unusual position of the viscera was found had reached the advanced age of eighty-six, and had been a remarkably powerful man in his youth, as I learned from himself and some others who had known him for many years.—*London Lancet.*

